# Preventive effects of betulinic acid on streptozotocinnicotinamide induced diabetic nephropathy in male mouse

**DOI:** 10.15171/jnp.2016.24

**Published:** 2016-08-03

**Authors:** Akram Ahangarpour, Ali Akbar Oroojan, Layasadat Khorsandi, Razieh Shabani, Shahnaz Mojaddami

**Affiliations:** ^1^Health Research Institute, Diabetes Research Center, Department of Physiology, Ahvaz Jundishapur University of Medical Sciences, Ahvaz, Iran; ^2^Department of Physiology, Student Research Committee of Ahvaz Jundishapur University of Medical Science, Ahvaz, Iran; ^3^Cell and Molecular Research Center, Department of Anatomical Sciences, Faculty of Medicine, Ahvaz Jundishapur University of Medical Sciences, Ahvaz, Iran; ^4^ 4Department of Physiology, Student Research Committee of Ahvaz Jundishapur University of Medical Science, Ahvaz, Iran

**Keywords:** Betulinic acid, Diabetic nephropathy, Metformin, Diabetes mellitus

**Implication for health policy/practice/research/medical education:**

Betulinic acid has a beneficial effect on diabetic nephropathy by improve plasma albumin, BUN, Cr and renal histology changes during diabetic situation. Therefore, the administration of betulinic acid might be preventing agent for nephropathy through the diabetic situation.

## 1. Introduction

Diabetes mellitus is a metabolic disorder that characterized by chronic hyperglycemia, hypertension, dyslipidemia, microalbuminuria and inflammation. Furthermore, there are many vascular complications associated with this disease such as retinopathy, neuropathy and nephropathy. Diabetic nephropathy is the main reason of 25%-40% end-stage renal disease. Diabetic nephropathy is defined as a progressive decline in glomerular filtration rate, accompanied by proteinuria (1). About 30% of people with newly diagnosed type 2 diabetes mellitus have abnormally high urine albumin levels, and about 75% of them have microalbuminuria and 25% showed obvious diabetic nephropathy (2). Blood urea nitrogen (BUN) and creatinine (Cr) are the simplest way to monitor kidney function. These substances are not excreted normally in renal disease, and accumulate in the body thus causing an increase in blood levels of urea Cr (3). Also, some study indicates that diabetic nephropathy induced by streptozotocin-nicotinamide (STZ-NA) can increase serum Cr level and developed renal progressive histopathological changes (2). Histopathological diabetic kidney shows a thickening of glomerular basement membrane, mesangial expansion, inter-tubular fibrosis and glomerulosclerosis which leads to microalbuminuria, hyperfiltration, and plasma albumin decreases through the urine albumin increases which can be detected by normal urinalysis (4).

Betulinic acid (BA) (3β-hydroxy-lup-20(29)-en-28-oic acid) is a lupane-type pentacyclic triterpenoid and precursor of botulin, distributed in a variety of plants such as outer bark of the white-barked birch tree *Betulaalba*, *Diospyros* species, *Ziziphus* species, *Syzygium* species, *Sarracenia flava*, *Inga punctate* and *Vauquelinia corymbosa*. BA can employ in the treatment and management of diabetes and its complications. Moreover, hepatoprotective effects of BA were found in HepG2 and hepatic stellate cells through its anti-inflammatory and antioxidant activity (5). Acute renal injury is associated with higher plasma Cr levels and widespread damage of tubules or epithelial necrosis that BA can attenuate them in pretreated mice (6). Additionally, renal ischemia reperfusion (I/R) increases serum levels of BUN and Cr in rats. However, when BA was administered before I/R, these BUN and serum Cr levels elevations were depressed significantly (7).

## 2. Objectives

Given the knowledge of beneficial effects of BA on renal function, the aim of present study was designed to evaluate the preventive effects of BA on STZ-NA induced diabetic nephropathy in male mouse.

## 2. Materials and Methods

### 2.1. Animal preparation

In this experimental study, 60 adult male NMRI mice (20-25 g) were obtained from Ahvaz Jundishapur University of Medical Sciences (AJUMS) animal facility. Mice used in this study were treated in accordance with principals and guidelines on animals care of AJUMS with an ethics committee number (IR.AJUMS.REC.1395.97) and, kept at 20±4^°^C with a 12 hour light/12 h dark cycle. They received free access to tap water and commercial chow.

### 2.2. Experimental design

After one week animals acclimatization to the laboratory, type 2 diabetes induced by intraperitoneal (IP) injection of a single dose of STZ (65 mg/kg; dissolved in citrate buffer, pH 4.5) (Sigma-Aldrich, USA) 15 minutes after an IP administration of NA (120 mg/kg, dissolved in normal saline) (Sigma-Aldrich, USA) (8). Diabetes induced was confirmed by assaying blood glucose levels at 1 week after NA-STZ injection and the mice with blood glucose level more than 200 mg/dl were used in the following experiments (9). Since, it has been suggested that the development of diabetic nephropathy would be started at 3 weeks after STZ injection (2); BA (Sigma-Aldrich, USA) and metformin (Sigma-Aldrich, USA) were orally administered 2 weeks after confirmed diabetes induction for prevention of diabetic nephropathy. Hence, mice were divided into six groups (10 mice in each group): control, diabetes group, diabetic mice receiving BA (10, 20 and 40 mg/kg) (10), and diabetic mice receiving metformin (200 mg/kg) (11) as a standard diabetes drug.

Twenty-four hours after the last drug administrations, the animals were sacrificed under deep anesthesia by ether and plasma samples were obtained by cardiac puncture blood collection and centrifuging at 3500 rpm for 15 minutes. Then, all samples were transferred to the microtubes and, maintained at −20^°^C (12). Finally, albumin, BUN and Cr of plasma samples were measured by using an Autoanalyzer device (BT3000, Italy) and, biochemical assay kits (Pars Azmoon, Iran).

### 2.3. Histopathological assessment

After blood collection, all mice kidneys were removed and fixed in 10% formalin solution. Then, dehydrated in graded alcohol concentrations and, embedded in paraffin. Sections of 3 µm were prepared and, stained with hematoxylin and eosin (H&E). Six microscopy slides per animal were examined for assessment of histological changes such as brush border loss, tubular dilatation, vasodilatation and infiltration of leukocytes. The average percentage of each criteria was determined by dividing the number of tubules with a histologic criteria including brush border loss in a randomly microscopic field by the total number of tubules in the same field and the result multiplied by 100 (13). Four categories were graded to infiltration of inflammatory cells; normal (0), weak (1), moderate (2) or intense (3) and the averages were considered. Tubules and dilated vessels were assessed by using Motic Images Plus 2.0 image analysis software. For each slide the mean of 6 fields were calculated. Slides were read in a “blind” fashion (14).

### 2.4. Ethical issues

Prior to the experiment, the protocols were confirmed to be in accordance with the Guidelines of Animal Ethics Committee of Ahvaz Jundishapur University of Medical Sciences. This research was approved by ethical committee of Ahvaz Jundishapur University of Medical Sciences (ethics committee number IR.AJUMS.REC.1395.97).

### 2.5. Statistical assessment

The statistical analysis was carried out by using SPSS software as mean ± standard error of mean (SEM) with one-way analysis of variance (ANOVA) followed by post hoc least significant difference (LSD) tests. Moreover, the statistical significance level was set at *P*<0.05.

## 3. Results

### 3.1. Effect of BA on plasma albumin, BUN and Cr levels

**Figure 1:**
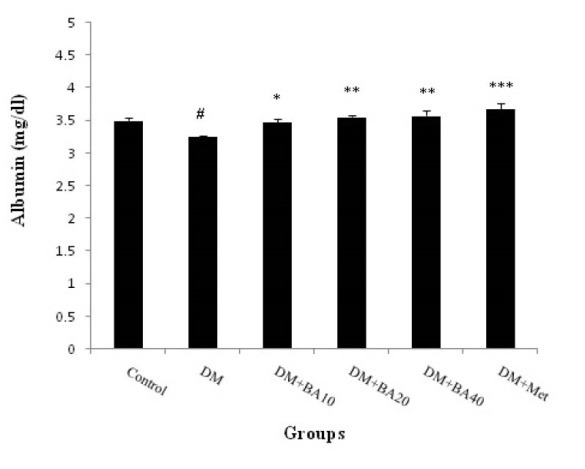
Effect of BA on plasma albumin levels. Data are shown as mean ± SEM, n = 10, (*P<0.05, **P<0.01, ***P<0.001) compared with the DM group, (#*P*<0.05) compared with the control group. BA: betulinic acid, DM: diabetes mellitus, Met: metformin.

**Figure 2:**
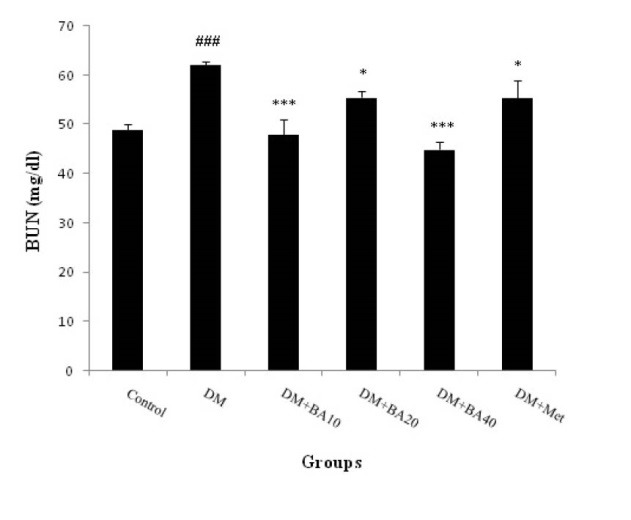
Effect of BA on BUN levels. Data are shown as Mean ± SEM, n = 10, (*P<0.05, ***P<0.001) compared with the DM group, (#*P*<0.05, ###*P*<0.001) compared with the control group. BA: betulinic acid, DM: diabetes mellitus, Met: metformin.

**Figure 3:**
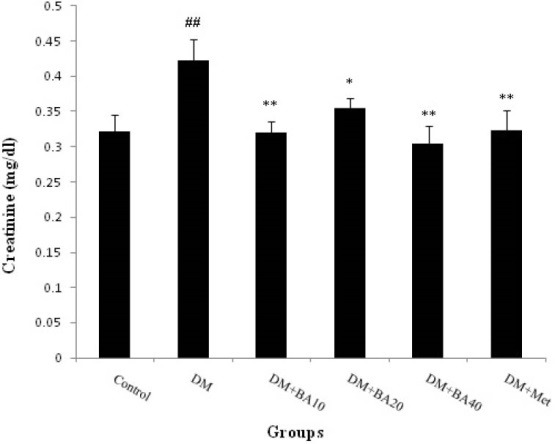
Effect of BA on plasma creatinine levels. Data are shown as mean ± SEM, n = 10, (*P<0.05, **P<0.01) compared with the DM group, (##*P*<0.01) compared with the control group. BA: betulinic acid, DM: diabetes mellitus, Met: metformin

The results showed that plasma albumin level decreased in diabetic mice when compared to control group (*P*<0.05) and increased in diabetic BA 10 mg/kg (*P*<0.05), BA 20, 40 mg/kg (*P*<0.01) and metformin (*P*<0.001) treated mice versus to diabetes group (Figure 1). Plasma levels of BUN increased in diabetes group (P<0.001) compared with control. Further, administration of BA 10 mg/kg (*P*<0.001), 20 mg/kg (*P*<0.05), 40 mg/kg (*P*<0.001) and metformin (*P*<0.05) showed a significant decrease in BUN in comparison with diabetes group (Figure 2). Plasma Cr levels as a diabetic nephropathy marker, increased in diabetes group compared to control (*P*<0.01) but, BA 10, 40 mg/kg, metformin (*P*<0.01) and BA 20 mg/kg (*P*<0.05) decreased this enhancement of plasma Cr levels (Figure 3).

### 3.2. Effect of BA on renal histopathology

**Figure 4:**
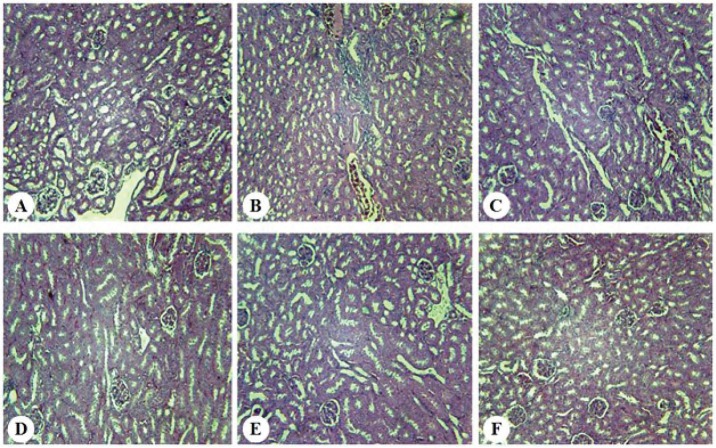
Effect of BA on renal histopathology. A: Control, B: Diabetes, C: Diabetes + BA 10 mg/kg, D: Diabetes + BA 20 mg/kg, E: Diabetes + BA 40 mg/kg, F: Diabetes + metformin. (H & E) (×400).

The results of renal histopathology indicated that renal normal appearance decreased in diabetic mice (*P*<0.05) and administration of BA or metformin improved this variable. Further, brush border loss, tubular dilation, and vasodilatation were increased in diabetes group (*P*<0.05) in comparison with other groups. Ultimately, it was revealed that infiltration of leukocytes has been occurred just in diabetic mice (*P*<0.05) and, this variable arrived at the control’s level after BA or metformin administration (Figure 4, Table 1).

**Table 1:** Effect of BA on scores changes of renal histology variable in normal and diabetic mice

**Groups**	**Histopathological criteria**				
	**Normal**	**Brush border loss**	**Tubular dilation**	**Vasodilatation**	**Infiltration of leukocytes**
Control	98.9%	0.6±0.9	0±0	0±0	0±0
Diabetes	74.2±9.2^#^	8.5±1.8^#^	2.5±0.6^#^	6.4±1.1^#^	2.6±0.4^#^
Diabetes+BA10	89.8±7.5^*^	3.9±0.5^*^	1.8±1.0^*^	1.7±0.3^*^	0±0^*^
Diabetes+BA20	91.4±8.3^*^	3.5±1.1^*^	1.6±0.2^*^	1.5±0.2^*^	0±0^*^
Diabetes+BA40	91.7±7.1^*^	3.8±0.9^*^	1.7±0.4^*^	1.6±0.3^*^	0±0^*^
Diabetes+ metformin	91.6±9.3^*^	3.6±0.8^*^	1.5±0.4^*^	1.4±0.6^*^	0±0^*^

Abbreviation: BA; betulinic acid. Data are shown as mean± SEM, n=10, (*P<0.05) when compared with diabetes group, (#*P*<0.05) when compared with control group.

## 5. Discussion

Present finding indicates that diabetes can induce nephropathy through the increase BUN, plasma Cr level and decrease in plasma albumin levels. Further, administration of BA can improve albumin reduction in a dose dependent manner. Also, BA affects more on BUN and Cr levels in low and high doses. Thus, it can be suggested that this agent may prevent diabetic nephropathy in 40 mg/kg dose of administration.

Serum albumin modifications have been reported in various diseases including types 1 and 2 of diabetes mellitus, renal failure, and end-stage renal disease. It is revealed that serum albumin levels are affected by various factors such as urinary albumin loss, albumin catabolism enhancement, and limitation of the maximal albumin production capacity by the liver. Further, hypoalbuminemia is a common finding in diabetic nephropathy that associated with chronic inflammation and severe oxidative stress. Diabetic nephropathy induced hypoalbuminemia has been attributed to urinary albumin excretion and occurs contrary to the great potential hepatic albumin production capacity (15).

It is widely known that the main reliable indicators to assess renal function are plasma BUN and Cr (16). Increase in BUN is occurring through the kidney dysfunction or damage. Increment of blood urea level concomitant with the blood sugar indicates that, hyperglycemia can lead to kidney damage (17). Plasma Cr is a more sensitive index of kidney function in comparison with urea level, because Cr fulfills most of the requirements for a perfect filtration marker. Also, measurement of Cr and urea plasma concentrations are the main tests for impairment assay of renal function through the type 2 diabetes mellitus and, it was detected that these factors are significantly higher in diabetic patients compared to non-diabetic (18). Some studies revealed an elevation of urea and plasma Cr and reduction of plasma albumin levels in diabetic rats (16,17,19). The results of Sharma et al study indicate that BUN and serum Cr level significantly increased in type 2 diabetic rats (20). Likewise, Viswanathan et al showed a significant serum albumin reduction in type 2 of diabetes (21). Hence, it can be suggested that, present finding is in agreement with previous studies.

Kidney structures are susceptible to hyperglycemia, and this metabolic change leads to organ damage via advanced glycation end products generation, abnormal protein kinase activation and raise of oxidative stress (22). In one study, BA exerted antidiabetic activities by reducing insulin resistance and potentiating β-cell mass and function. Several studies have shown that BA improves glucose metabolism and enhanced insulin-stimulated glucose uptake in the adipose tissue and liver of diabetic rats (23). Therefore, present nephroprotective findings may produce by antidiabetic and hypoglycemic effects of BA administration.

Histopathology in diabetic nephropathy is characterized by several modifications such as tubulointerstitial inflammation, brush border loss, and accumulation of extracellular matrix (24). As Jheng et al (25) study showed a remarkable loss of brush border or tubular dilation, the present results revealed a significant increase in brush border loss, tubular dilation, vasodilatation and infiltration of leukocytes after induction of diabetic nephropathy by STZ-NA. The study of Lingaraju et al indicated that BA utilization was successful in ameliorating the renal histopathological changes induced by an experimental model of murine polymicrobial sepsis and one of the proposed influence mechanisms of BA in these changes were its antioxidant activity (6). Hence, due to the similarity of the present results with the study of Lingaraju et al, it could be suggested that renal protective effect of BA has been occurred through its antioxidant properties, but future studies are required to clarify the exact mechanism of this event.

## 6. Conclusions

In conclusion, STZ-NA induced diabetes can lead to diabetic nephropathy by reducing plasma albumin level and enhancement of BUN, Cr and renal histopathology. On the other side, BA has a preventing effect on diabetic nephropathy by amelioration of plasma albumin, BUN, Cr and renal histology changes during diabetic situation.

## Authors’ contribution

AA and AAO conducted the research. AA, AAO and LK analyzed the data and prepared the pri­mary draft. AA, AAO, LK, RS and SM conducted the experimental measurements.

## Conflict of interests

The authors declared no competing interests.

## Funding/Support

This study is labeled Student Research Project No. 94S133, and was supported financially by the Student Research Committee of Ahvaz Jundishapur Medical Sciences University, Ahvaz, Iran.
